# Fetal cranial growth trajectories are associated with growth and neurodevelopment at 2 years of age: INTERBIO-21st Fetal Study

**DOI:** 10.1038/s41591-021-01280-2

**Published:** 2021-03-18

**Authors:** José Villar, Robert B. Gunier, Chrystelle O. O. Tshivuila-Matala, Stephen A. Rauch, Francois Nosten, Roseline Ochieng, María C. Restrepo-Méndez, Rose McGready, Fernando C. Barros, Michelle Fernandes, Verena I. Carrara, Cesar G. Victora, Shama Munim, Rachel Craik, Hellen C. Barsosio, Maria Carvalho, James A. Berkley, Leila Cheikh Ismail, Shane A. Norris, Eric O. Ohuma, Alan Stein, Ann Lambert, Adele Winsey, Ricardo Uauy, Brenda Eskenazi, Zulfiqar A. Bhutta, Aris T. Papageorghiou, Stephen H. Kennedy

**Affiliations:** 1 Nuffield Department of Women’s & Reproductive Health, University of Oxford, Oxford, UK; 2 Oxford Maternal & Perinatal Health Institute, Green Templeton College, University of Oxford, Oxford, UK; 3 Center for Environmental Research and Children’s Health, School of Public Health, University of California, Berkeley, Berkeley, CA, USA; 4 SAMRC Developmental Pathways For Health Research Unit, Department of Paediatrics & Child Health, University of the Witwatersrand, Johannesburg, South Africa; 5 Health, Nutrition & Population Global Practice, World Bank Group, Washington, DC, USA; 6 Centre for Tropical Medicine and Global Health, Nuffield Department of Medicine Research building, University of Oxford Old Road Campus, Oxford, UK; 7 Shoklo Malaria Research Unit, Mahidol-Oxford Tropical Medicine Research Unit, Faculty of Tropical Medicine, Mahidol University, Mae Sot, Thailand; 8 Faculty of Health Sciences, Aga Khan University, Nairobi, Kenya; 9 Centre for Tropical Medicine and Global Health, Nuffield Department of Medicine, University of Oxford, Oxford, UK; 10 Programa de Pós-Graduação em Saúde e Comportamento, Universidade Católica de Pelotas, Pelotas, Brazil; 11 Faculty of Medicine, Department of Paediatrics, University of Southampton, Southampton, UK; 12 Programa de Pós-Graduação em Epidemiologia, Universidade Federal de Pelotas, Pelotas, Brazil; 13 Department of Obstetrics and Gynaecology, Division of Women and Child Health, Aga Khan University, Karachi, Pakistan; 14 KEMRI-Coast Centre for Geographical Medicine and Research, University of Oxford, Kilifi, Kenya; 15 Department of Obstetrics & Gynaecology, Faculty of Health Sciences, Aga Khan University Hospital, Nairobi, Kenya; 16 KEMRI/Wellcome Trust Research Programme, Nairobi, Kenya; 17 Clinical Nutrition and Dietetics Department, University of Sharjah, Sharjah, United Arab Emirates; 18 SAMRC Developmental Pathways For Health Research Unit, Department of Paediatrics & Child Health, University of the Witwatersrand, Johannesburg, South Africa; 19 Maternal, Adolescent, Reproductive & Child Health (MARCH) Centre, London School of Hygiene & Tropical Medicine, London, UK; 20 Department of Psychiatry, University of Oxford, Oxford, UK; 21 MRC/Wits Rural Public Health and Health Transitions Research Unit (Agincourt), School of Public Health, Faculty of Health Sciences, University of the Witwatersrand, Johannesburg, South Africa; 22 Department of Nutrition and Public Health Interventions Research, London School of Hygiene & Tropical Medicine, London, UK; 23 Center for Global Child Health, Hospital for Sick Children, Toronto, Ontario, Canada

## Abstract

Many observational studies and some randomized trials demonstrate how fetal growth can be influenced by environmental insults (for example, maternal infections)^1^ and preventive interventions (for example, multiple-micronutrient supplementation)^2^ that can have a long-lasting effect on health, growth, neurodevelopment and even educational attainment and income in adulthood^3^. In a cohort of pregnant women (*n* = 3,598), followed-up between 2012 and 2019 at six sites worldwide^4^, we studied the associations between ultrasound-derived fetal cranial growth trajectories, measured longitudinally from <14 weeks’ gestation, against international standards^5,6^, and growth and neurodevelopment up to 2 years of age^7,8^. We identified five trajectories associated with specific neurodevelopmental, behavioral, visual and growth outcomes, independent of fetal abdominal growth, postnatal morbidity and anthropometric measures at birth and age 2. The trajectories, which changed within a 20-25-week gestational age window, were associated with brain development at 2 years of age according to a mirror (positive/negative) pattern, mostly focused on maturation of cognitive, language and visual skills. Further research should explore the potential for preventive interventions in pregnancy to improve infant neurodevelopmental outcomes before the critical window of opportunity that precedes the divergence of growth at 20-25 weeks’ gestation.

The literature on the association between events during fetal life and postnatal growth and neurodevelopment into childhood is replete with limitations. For example, gestational age is rarely estimated accurately^5^; hence, the precise relationship between an intrauterine insult, such as maternal infection, and faltering growth is difficult to determine. Similarly, the severity of fetal growth restriction (FGR) cannot be determined unless serial ultrasound scans are performed, and birth weight alone as a proxy measure for the totality of intrauterine growth is insufficient^9^. In addition, most large-scale pregnancy and newborn epidemiological data are based on retrospective analyses of routinely collected, but rarely standardized, medical records^10^. In this study, we investigated associations among fetal cranial growth trajectories, measured by serial ultrasound scans from <14 weeks’ gestation, against international standards^5,6^ and growth and neurodevelopment up to the key milestone age of 2 years. We sought to identify windows of opportunity for interventions during pregnancy to improve health and neurodevelopmental outcomes.

## Novel trajectories identified based on fetal cranial growth

**Fig. 1:**
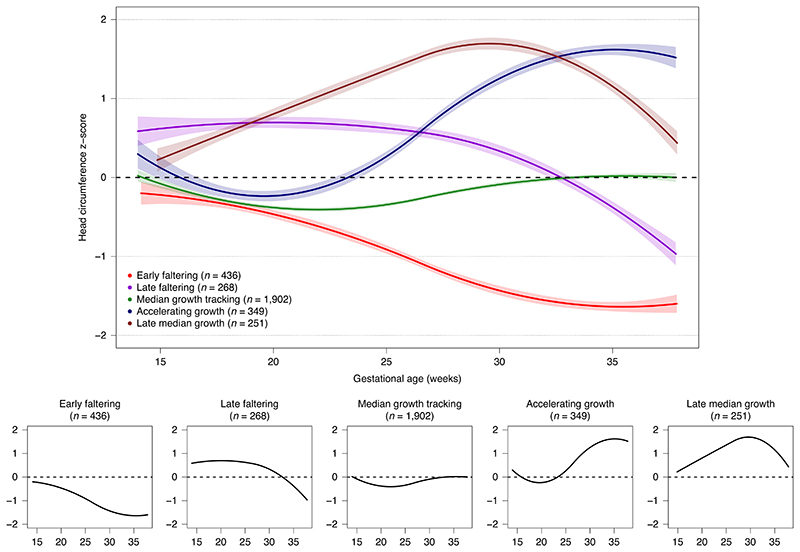
Fetal cranial growth trajectories in the INTERBIO-21st Study.

The best-fitting models of fetal cranial growth had five trajectories (Fig. 1). The average posterior probabilities for membership in the selected group ranged from 0.67 to 0.75; the odds of correct classification ranged from 3.8 to 6.8 (Supplementary Table 1); and the model with five groups had the lowest Bayesian information criterion (BIC) (Supplementary Table 2), indicating good model fit^11^. More than half (*n* = 1,902) of the fetuses had a steady growth trajectory throughout pregnancy, close to the INTERGROWTH-21st 50th centile^5^. They comprised the reference group for subsequent analyses (median growth tracking (MGT)). The second trajectory (early faltering (EF), *n* = 436) displayed growth faltering throughout pregnancy. The third (late faltering (LF), *n* = 268) showed higher (close to +0.5 of s.d.) but parallel growth to the MGT trajectory through the second trimester, with growth starting to falter by the early third trimester. The fourth (accelerating growth (AG), *n* = 349) showed growth close to the 50th centile through the second trimester and accelerated growth during the third trimester. The fifth (late median growth tracking (LMGT), *n* = 251) had accelerated growth after 15 weeks’ gestation and then a ‘normalizing’ growth rate in the third trimester (Fig. 1). The observed trajectories, like all models, are just approximations of a more complex reality and should be interpreted based on the overall pattern, not on small portions of the curves.

## Development, growth and morbidity at age 2

The INTERGROWTH-21st Neurodevelopmental Assessment (INTER-NDA) cognitive, fine motor, gross motor, negative behavior and positive behavior scores^12,13^ of the total study sample were close to the median of the international INTER-NDA standards and slightly below the median of international standards for language-scaled scores and the positive behavior scale^8^. A high percentage of children scored in the ‘clinical’ range (>97th centile) for the Child Behavior Checklist’s (CBCL) attentional problems (16.8%) and emotional reactivity (19.8%) scale^14^. Scaled total scores were 3.9 ± 2.3 for attentional problems and 5.7 ± 3.6 for emotional reactivity.

Based on the Cardiff scale^15^, 16% of children had visual acuity scores and 11% had contrast sensitivity scores below the norms for 2 year olds (logMAR 0.4–0.1 for visual acuity and 33.3–100% for contrast sensitivity), compared to the 10% expected prevalence in the INTERGROWTH-21st population^16^. The median ages of attaining the World Health Organization (WHO) milestones^17^ were within the normal ranges: from 6 months for sitting without assistance to 12.8 months for walking without assistance. Overall, children’s height at age 2 was about one third of an s.d. less than the WHO Child Growth Standard (mean *z*-score = –0.34 ± 1.3) but close to the mean *z*-score for weight and head circumference (HC)^7^. The morbidity episodes reported at ages 1 and 2 showed moderate to high rates of clinical conditions, compared to the INTERGROWTH-21st population^18^, reflecting our strategy of enriching for peri-conceptual risk characteristics at enrollment known to lead to suboptimal size, morbidity and/or neurodevelopment delay (Supplementary Table 3).

## Developmental and growth outcomes at age 2 in relation to fetal cranial growth trajectories

Supplementary Table 4 presents mean (s.d.) values for neurodevelopmental domains and visual scores as well as 2-year anthropometric measures according to trajectory. There was a clear pattern of lower neurodevelopmental and vision scores in the EF trajectory, with the LF trajectory at an intermediate level. The AG and LMGT trajectories had higher but similar scores than the MGT and the two faltering trajectories. There was no difference in the age at walking alone among trajectories.

**Fig. 2:**
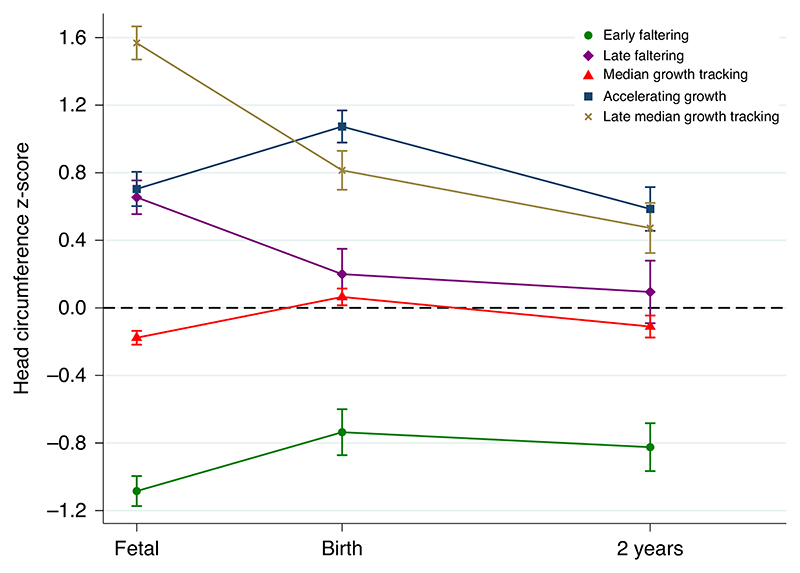
Trajectories of head circumference *z*-scores from prenatal measurements through 2 years of age, stratified by fetal cranial growth trajectories Fetal measures are from the ultrasound visit closest to 27 weeks’ gestation. Error bars represent the 95% confidence intervals of the means.

Similar patterns were observed for anthropometric measures at birth^19^: for example, the newborn HC *z*-score for the EF trajectory was -0.7 of the s.d. (Fig. 2). The opposite effect was seen in the AG and LMGT trajectories, with approximately 1-s.d. increase in HC at birth. The LF and MGT trajectories were very close at age 2.

To understand how consistent the phenotypic patterns were from mid-pregnancy to age 2, we compared the *z*-scores of fetal HC at a mean gestational age of 27.5 weeks with those for HC at birth and age 2. The phenotypic differences persisted (that is, tracking their fetal path), but, from birth onwards, there was a trend to ‘normalization’ with age: the mean infant HC values for the five trajectories were within ± 0.8 *z*-scores by age 2, with the LF trajectory very close to the MGT trajectory at about the 50th centile (Fig. 2).

**Fig. 3:**
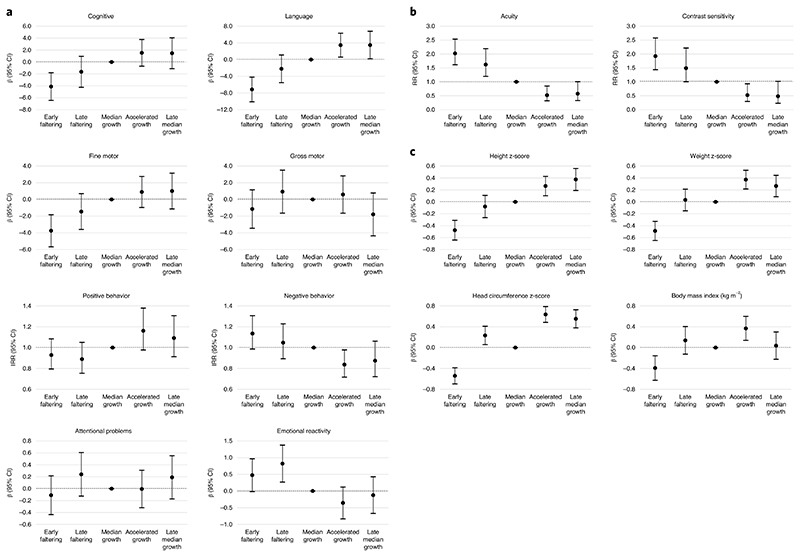
Changes at 2 years of age associated with fetal cranial growth trajectories in the INTERBIO-21st Study Child neurodevelopmental outcomes. For cognitive, language, motor and positive behavior outcomes, higher scores represent better outcomes; for negative behavior, attentional problems and emotional reactivity, higher scores represent worse outcomes. **b**, Child vision. Relative risks > 1 refer to an increased risk of poor visual performance; relative risks < 1 refer to a reduced risk of problems. **c**, Child growth: change in *z*-scores. Median growth tracking trajectory is the reference group. Models include maternal education and age at birth, preterm birth and smoking in pregnancy, and child sex and age at assessment.

In the models adjusted for sex, age at assessment, preterm birth, maternal smoking, age and education, relative to the reference MGT trajectory, the EF trajectory scored poorest on the cognitive (β = -4.12, 95% confidence interval (CI) = -6.45, -1.80), language (β = -7.16, 95% CI = -10.12, -4.20) and fine motor (β = -3.75, 95% CI = -5.67, -1.83) domains of the INTER-NDA (Fig. 3 and Table 1). These children also showed the highest risk for poor vision outcomes—that is, high logMAR for visual acuity (relative risk (RR) = 2.02, 95% CI = 1.61, 2.53) and high percentage for contrast sensitivity (RR = 1.92, 95% CI = 1.44, 2.58). In addition, their growth was diminished at 2 years of age, with *z*-scores for height (β = -0.48, 95% CI = -0.64, -0.31), weight (β = -0.49, 95% CI = -0.65, -0.33) and HC (β = -0.54, 95% CI = -0.70, -0.39), respectively.

However, the LF trajectory, adjusting for the same variables, showed intermediate patterns, with CIs crossing the null effect relative to the reference group; the sole difference, among the eight domains evaluated, was higher levels of emotional reactivity (β = 0.82, 95% CI = 0.27, 1.38) on the Child Behavior Questionnaire. Although less affected than children in the EF trajectory, these children had a higher risk of poorer visual acuity (RR = 1.62, 95% CI = 1.20, 2.19) and contrast sensitivity (RR = 1.49, 95% CI = 1.00, 2.22) (Fig. 3 and Table 1). Compared to the reference trajectory, their height, weight and body mass index (BMI) were similar, but their HC *z*-scores were slightly higher (β = 0.23, 95% CI = 0.06, 0.41) (Fig. 3 and Table 1).

Conversely, children in the AG trajectory scored higher on the INTER-NDA language domain (β = 3.45, 95% CI = 0.59, 6.32), with a decreased risk of poorer visual acuity (RR = 0.52, 95% CI = 0.32, 0.85) and contrast sensitivity (RR = 0.52, 95% CI = 0.29, 0.93). These children had increased *z*-scores for height (β = 0.27, 95% CI = 0.10, 0.43), weight (β = 0.37, 95% CI = 0.22, 0.53) and HC (β = 0.64, 95% CI = 0.48, 0.79) (Fig. 3 and Table 1).

Finally, children in the LMGT trajectory showed better performance on the INTER-NDA language domain (β = 3.48, 95% CI = 0.18, 6.78). These children were also at marginally lower risk of developing poorer visual acuity (RR = 0.57, 95% CI = 0.33, 1.01) and contrast sensitivity (RR = 0.48, 95% CI = 0.23, 1.02). They also had increased *z*-scores for height (β = 0.38; 95% CI = 0.19, 0.56), weight (β = 0.27; 95% CI = 0.09, 0.44) and HC (β = 0.55; 95% CI = 0.38, 0.72) (Fig. 3 and Table 1).

We evaluated correcting our results for multiple comparisons using the Benjamini-Hochberg false discovery rate correction to control for type 1 error rate at <0.05, and all of our findings with the EF group and all groups related to 2-year growth outcomes remained highly statistically significant^20^. We further estimated models that simultaneously included both fetal HC and abdominal circumference (AC) trajectories to evaluate the effect of HC independently of other fetal ultrasound measures. Results were similar to those from the separate HC and AC trajectory models, supporting the hypothesis that associations of HC with growth and development at age 2 are independent of other fetal growth parameters.

We conducted sensitivity analyses excluding preterm births, neonatal intensive care unit (NICU) admissions, serious infant morbidity and frequent infections or hospitalizations, and the results were similar. Sensitivity analyses were also performed including possible outliers of the 2-year anthropometric measures (*n* = 5): no relevant changes were observed, except that the HC *z*-score association with the EF trajectory was weaker. In sensitivity analyses excluding those children missing one or more questions from the INTER-NDA, the associations were similar or slightly stronger for all domains and trajectories, except that the relationship between the AG trajectory and cognitive domain became stronger and statistically significant (β = 2.29; 95% CI = 0.19, 4.39).

In an exploratory analysis, we did not observe any specific pattern indicative of a differential role for the length of breastfeeding on the AG, LF or LMGT trajectories (Supplementary Table 5). However, there was a consistent protective effect of breastfeeding for ≥7 months on the cognitive, language, fine motor, negative behavior, emotional reactivity, visual acuity and contrast sensitivity domains among children in the EF trajectory compared to children in the MGT trajectory, although the tests for interaction only reached statistically significant levels (*P* < 0.01) for the fine motor, emotional reactivity and contrast sensitivity domains. We adjusted these models for child sex, age at developmental assessment, preterm birth, maternal age and education and smoking during pregnancy. There was no effect on height or HC at age 2, but longer breastfed children in the EF trajectory improved their BMI by that age (*P* = 0.06) (Supplementary Fig. 1).

We have demonstrated that five fetal trajectories of cranial growth, identified from ultrasound-derived, longitudinal, fetal HC measures, are associated with specific neurodevelopmental, behavioral, visual and growth outcomes at age 2 (ref. ^21^). Although a relationship between behavioral traits and serial measures of HC in healthy infants was described previously^22^, we have introduced, to our knowledge for the first time, a phenotypic classification of fetal cranial growth with functional relevance to important early childhood outcomes that influence cognition, language, educational achievement and productivity in later life^23^.

These effects are independent of 1) other fetal growth measures (for example, AC); 2) gestational age at birth/preterm status; 3) neonatal and child morbidity; 4) HC growth from birth to age 2; 5) maternal age and level of formal education, smoking and demographic characteristics; 6) gross infant motor markers (for example, age at first walking); and 7) child sex and exact chronological age at developmental assessment, which strongly suggest a direct effect of differential fetal cranial growth patterns on early child development. Notably, the growth variations identified occur well within the normal range of ±2 s.d. of fetal HC growth^5^ and ±1 s.d. of the WHO standard for HC at age 2 (ref. ^7^), indicating that the association is not the result of extreme growth trajectories or specific study sites contributing the majority of fetuses but, rather, part of the variability among the total study sample. However, we also acknowledge that, as with all biological studies, there was likely random measurement error related to HC, which would likely result in non-differential misclassification of participants into trajectories and bias results toward the null, resulting in an underestimate of the strength of the associations observed^24^.

We identified a window between 20 and 25 weeks’ gestation during which cranial growth either tracked the INTERGROWTH-21st 50th centile or its trajectory changed due to growth accelerations/deaccelerations (Fig. 1). This is a critical time for brain development when the number of lifetime available neocortical neurons is being determined through neuronal pruning^25^. The diverging patterns are likely related to factors present before this window or even pre-pregnancy.

The EF trajectory is the most striking example of cranial growth faltering, with a strong negative effect on cognitive, fine motor, language and visual development at age 2. Postnatal cranial growth also remained affected in this group: by age 2, the *z*-score was —0.8 of the WHO standard (Fig. 2). Conversely, the two trajectories with accelerated growth in this time window (AG and LMGT) had a very similar, strong positive association with language and visual development at age 2. This effect was present despite differences in late pregnancy cranial growth patterns, reinforcing the likelihood that the effect originates in the first half of pregnancy. Furthermore, the LF trajectory, although affected in the vision and emotional reactivity domains (that is, a negative effect can be produced in the early third trimester), remained close to the reference trajectory with limited effects on development or growth (Figs. 1-3).

In short, these results strongly support the concept that, between 20 and 25 weeks’ gestation, neurodevelopment (using fetal cranial growth as a proxy) is associated with a consistent, mirror (positive/negative) pattern mostly focused on maturation of cognitive, language and visual skills, independent of major confounding variables, such as fetal AC growth, postnatal morbidity and anthropometric measures at birth and age 2.

To our knowledge, our study is unique because 1) it was based on following a prospective, multinational cohort exposed to multiple risk factors; 2) pregnancies were accurately dated in the first trimester; and 3) postnatal growth was monitored according to WHO recommendations. Data collection was standardized, which allowed adjustment for possible confounding variables across sites using the same variables and definitions. Hence, the adjusted analyses reflecting independent associations can be considered robust. However, whether the trajectories identified can be used as a screening tool at the population or individual level will require considerably more research.

One limitation of our analytical strategy was that we did not explore early pregnancy etiological or risk factors (for example, bio-markers of maternal nutritional status) known to be associated with altered fetal growth. To incorporate such a detailed analysis, given our focus on the association between actual fetal growth trajectories and neurodevelopment, would have required including considerable additional data. We also lacked parental and sibling developmental profiles, which might have helped the adjusted analysis, as well as the family developmental risk that can act as an effect modifier of the observed association.

In conclusion, we propose three suggestions for translating the biological evidence presented here into clinical and public health actions. First, it is highly unlikely that a ‘silver bullet’ will ever be identified to prevent or treat FGR because it is such a heterogeneous syndrome. Second, previously tested interventions in pregnancy might have failed to improve neurodevelopmental outcomes because they were implemented after the critical window of opportunity for intervention has passed, or the sample size was too small to show effects on a specific HC trajectory. Lastly, there is no substitute for the strategy of initiating ante-natal care as early as possible for pregnant women worldwide.

## Methods

### Study sites

Phase II of the INTERGROWTH-21st Project (the INTERBIO-21st Fetal Study) was conducted between 2012 and 2019 at six sites: Pelotas (Brazil), Nairobi (Kenya), Karachi (Pakistan), Soweto (South Africa), Mae Sot (Thailand) and Oxford (UK). The sites in Pelotas, Nairobi and Oxford also participated in Phase I of the INTERGROWTH-21st Project, which showed consistent similarities in fetal growth and newborn size at birth and similar patterns of skeletal growth, neurodevelopment and associated behaviors at age 2, in a multinational cohort of healthy, educated and well-nourished women, living in clean environments and at low risk of adverse maternal and perinatal outcomes^5,9,16^.

Detailed information about each study site was previously published^4,16^. In brief, Pelotas (Brazil) is the third most populous city in the state of Rio Grande do Sul, with 350,000 inhabitants and 4,000 births per year, nearly all of which take place in the city’s four maternity hospitals. Nairobi (Kenya): More than 4,000 births per year in the relatively wealthy Parklands suburb of Nairobi, a geographically delimited urban area, take place in three hospitals, the largest of which, the Aga Khan University Hospital (AKUH), participated in the study. Nairobi is a non-endemic malaria area. The ante-natal HIV prevalence in this hospital is 1%. Karachi (Pakistan): The AKUH, Karachi, is a not-for-profit teaching hospital, serving a range of socio-economic groups in the largest city in Pakistan, with an estimated population of approximtely 20 million people. The hospital has 5,800 births per year; one-third are high risk, referred from four secondary care hospitals (combined total >15,000 births per year). Soweto (South Africa): Chris Hani Baragwanath Academic Hospital, which is attached to the University of the Witwatersrand, Johannesburg, is the only government hospital serving a population of approximately 1.3 million people. It has approximately 17,000 births per year, 75% of which are medium to high risk; this represents 53% of all births in Greater Soweto. The HIV prevalence is 29%. Mae Sot (Thailand): The Shoklo Malaria Research Unit is a Mahidol-Oxford Research Unit field station for studying malaria (including in pregnancy) among the 130,000 people living in refugee camps on the Thai-Myanmar border. Three of its field sites, with a total of approximately 2,000 births per year, participated in the study: two (Wang Pha and Mawker Thai) are clinics for migrants, and the third (Maela camp) is the largest refugee camp along the border. Oxford (UK): The John Radcliffe Hospital covers approximately 75% of more than 8,000 births per year in Oxfordshire, which has a population of more than 650,000 people that includes a large proportion of young, middle-class, well-educated and professional families.

### Participants

In the INTERBIO-21st Fetal Study, we enrolled 3,598 women who initiated ante-natal care before 14 weeks’ gestation, irrespective of their risk profile for adverse pregnancy/neonatal outcomes, and monitored their pregnancies to delivery. Thereafter, the health, growth and development of their children were monitored up until age 2. The only inclusion criteria at study entry were maternal age ≥18 years, BMI <35 kg m^-2^ (to facilitate ultrasound scanning of the fetus), natural conception and singleton pregnancy. All other women were eligible to participate after giving written informed consent. Each site’s contribution ranged from 12.4% (Pelotas, *n* = 397) to 20.2% (Oxford, *n* = 647). The other sites contributed 17.3% (Soweto, *n* = 554), 15.7% (Karachi, *n* = 502), 16.5% (Mae Sot, *n* = 530) and 18% (Nairobi, *n* = 576), respectively.

### Pregnancy data

Data collection relating to pregnancy followed the same strategy as in the Fetal Growth Longitudinal Study (FGLS) of Phase I of the INTERGROWTH-21st Project^5^. In brief, a comprehensive set of variables was obtained prospectively using data collection forms and an electronic data entry system specifically developed for the study (www.interbio21.org.uk). Baseline information included demographic and nutritional characteristics, medical, gynecological and obstetric history and current pregnancy conditions. Pregnancy follow-up information included routine standard ante-natal care variables, current health, use of supplements or medication and referral to another level of care or hospital. Pregnancy morbidity was also recorded during follow-up, including diagnoses such as gestational diabetes, pregnancy hypertension, pre-eclampsia, vaginal bleeding, preterm premature rupture of membranes, preterm labor, severe vomiting requiring hospitalization, eclampsia/HELLP syndrome (hemolysis, elevated liver enzymes and low platelet count), rhesus disease, abruptio placentae, clinical chorioamnionitis, mode of delivery and any pregnancy-related condition or infection requiring treatment (for example, malaria).

### Fetal ultrasound scans

The ultrasound methods used to estimate gestational age, as well as the training, standardization and quality control processes, were described previously^5^. In brief, crown-rump length was measured < 14^+0^ weeks’ gestation, with an angle of insonation as close as possible to 90°, in a mid-sagittal view of the horizontal fetus in a neutral position filling at least 30% of the screen. The calipers were placed on the outer borders of the head and rump, and gestational age was estimated using the INTERGROWTH-21st standards for pregnancy dating^6^. When the date of the last menstrual period was available, this was also recorded.

After the dating ultrasound scan, the women were seen every 5 ± 1 weeks until delivery (that is, 14-18, 19-23, 24-28, 29-33, 34-38 and 39-42 weeks’ gestation), as in the FGLS. Identical ultrasound equipment (Philips HD-9, Philips Ultrasound, with curvilinear abdominal transducers C5-2, C6-3, V7-3) was used at each site. Of the 3,598 women enrolled, 3,206 had at least three fetal ultrasound scans between <14 and 37 weeks’ gestation.

At each visit from 14 weeks’ gestation onwards, the fetal HC was obtained in the transthalamic plane, placing the calipers on the outer border of the skull, using both the ellipse facility and two perpendicular diameters. We selected HC as an ultrasound marker of fetal cranial growth because 1) it is widely used in routine obstetric practice with complementary skeletal growth markers and avoids the measurement errors associated with biometric ratios; 2) measurement was standardized across study sites; 3) it relates directly to brain and cerebellar growth; and 4) importantly for a postnatal follow-up study, HC in childhood is the same measure.

The AC was obtained in an axial plane, with the umbilical vein in the anterior third of the fetal abdomen (at the level of the portal sinus) and the stomach bubble visible. Both the ellipse facility and the two diameters method were used, placing the calipers on the outer border of the body outline (skin covering). All measurements were taken three times from three separately generated ultrasound images. To avoid expected value bias, the ultrasound machines were specially adapted to ensure that the ultrasonographers were ‘blinded’ to the actual values, which were transferred electronically and available only after the examination was completed. In the present analysis, we focused on HC, because it is a reliable proxy for cranial growth; the use of a single measure, rather than a summary measure such as estimated fetal weight, is associated with a lower measurement error, and the same measurement can easily be made longitudinally in postnatal life.

The ultrasonographers at each site were selected on the basis of their technical expertise, motivation, reliability and ability to speak the local language(s). Through rigorous training, they gained theoretical knowledge and familiarity with the study protocol, operations manual, data collection and quality control measures. We also conducted centralized, hands-on training, and the Oxford-based Ultrasound Quality Control Unit regularly carried out site-specific standardization procedures to ensure proper use of the ultrasound equipment and protocol adherence. Quality control was maintained throughout the study by taking a random 10% sample of all ultrasound images and assessing their quality using a validated scoring system.

### Newborn anthropometric measures

The anthropometric measurement protocols and quality control procedures were identical to those used in the FGLS^19^. In brief, newborn measures were ideally obtained within 12 h of birth (and no later than 24 h), using the same equipment at all sites: an electronic scale (Seca) for birth weight (sensitivity of 10 g up to 20 kg) and a specially designed Harpenden Infantometer (Chasmors) for recumbent length. The equipment was selected for accuracy, precision and robustness, as reported in previous studies^19^, and calibrated twice weekly. HC was measured using a metallic non-extendable tape (Chasmors).

All lead anthropometrists were trained centrally to measure newborns according to the study protocol, and, in turn, they trained the local anthropometrists who were standardized at regular intervals; all training materials were based on the original WHO protocols. The Anthropometric Standardisation Unit based in Oxford regularly monitored the performance of all staff. The quality control measures required anthropometrists to take and record all measures twice independently and compare their values with the maximum allowable differences: newborn weight, 50 g; length, 7 mm; and HC, 5 mm. If the difference between the two measures exceeded these values, then both observers independently repeated that measurement a second time and, if necessary, a third time^19^.

### Infant follow-up

Across all sites, standardized clinical care and feeding practices were implemented based on protocols developed by the INTERGROWTH-21st Neonatal Group (www.intergrowth21.org.uk). Exclusive breastfeeding up to 6 months and appropriate nutritional support for infants born preterm were promoted during and after pregnancy as recommended^26^.

Detailed information was obtained from the mother at age 1 and 2 about the infant’s health, severe morbidities, hospitalizations, length of breastfeeding, timing of the introduction of solid food, age at weaning, feeding practices and food intake, using specially produced forms (www.intergrowth21.org.uk). The proportion of infants receiving breast milk and vitamin and mineral supplements and those following a special diet were estimated at age 1 and 2 (refs. ^27,28^).

Similarly, at age 1 and 2, the infants’ weight, length, HC, mid-upper arm circumference, triceps skinfold thickness and subscapular skinfold thickness were measured following WHO protocols^29^, and their age- and sex-specific *z*-scores and centiles were compared to the WHO Child Growth Standards^17^. Corrected age was used for those infants born preterm^30^. We used linear and cranial growth at age 2 as the main anthropometric outcomes because median adult height in healthy populations is close to double the median height at that age, and, by then, approximately 60% of adult HC has been achieved^18,31^. Thereafter, children tend to follow the same growth trajectory with limited movement across centiles^32^. Thus, these anthropometric measures, as indicators of general nutrition at age 2, are strongly predictive of later attained height, development and human capital^33^. Unfortunately, parental head size and shape were not obtained, nor was neonatal head shape other than those classified as birth defects, which were excluded from the analysis sample.

We analyzed the pre- and postnatal anthropometric measures expressed as *z*-scores of the corresponding international standards. We chose to use these standards because they meet the WHO requirement to monitor human growth^34^, and they provide information on postnatal growth and development to estimate *z*-scores, based on the same population, across the period from early pregnancy to 2 years of age. Such a degree of standardization for monitoring growth longitudinally has seldom been achieved in this field.

During the infant follow-up, there were 21 deaths and 439 losses to follow-up; in addition, 279 children were already more than 1 year old when assessment began. Thus, 2,443 children were assessed at 1 year (85% of those eligible). There were 198 children who attended the 2-year, but not the 1-year, visit. Overall, 2,183 children completed the 2-year follow-up (76% of those eligible) (Supplementary Fig. 2).

The mean age of mothers of children seen at age 2 (*n* = 2,183) was 29.4 ± 5.2 years; their BMI at recruitment was 24.8 kg m^-2^. Most (88%) were married or co-habiting, 42% were nulliparous and 43% had at least a university education; 71% worked outside the home before pregnancy. Very few mothers smoked or drank alcohol during pregnancy (4.1% and 2.2%, respectively). Fifty-two percent of all newborns were male, and 42% were delivered by Caesarean section; about 9% were preterm, with less than 2% very preterm (<34 weeks’ gestation).

Similar baseline patterns were observed between the total sample that contributed measures to construct the fetal trajectories (all participants, *n* = 3,206) and the sample participating in the 2-year follow-up component (*n* = 2,183). Women completing the 2-year follow-up were very similar to the full cohort, only they were marginally better educated, and their babies had lower very preterm and NICU admission rates. Infants lost to follow-up were also similar at birth to those completing the 2-year follow-up, except that they were more likely to be preterm and have had a NICU admission (Supplementary Tables 6 and 7).

### Neurodevelopment assessment

We assessed neurodevelopment at age 2 using the INTER-NDA, an international, multicultural, standardized, psychometric tool, targeted at children aged 22–30 months, which measures multiple dimensions of early development using a combination of directly administered, concurrently observed and caregiver-reported items^12^. It was designed to be implemented by non-specialists across international settings^13^ and includes a reduced number of culture-specific items comprising six domains measuring cognition, language (expressive and receptive), fine and gross motor skills and positive and negative behavior, in an assessment time of 15 min on average. The INTER-NDA has been validated against the Bayley Scales of Infant Development III edition, showing good to moderate agreement^13^, and has shown good levels of inter-rater (*k* = 0.70; 95% CI: 0.47–0.88) and test/re-test reliability (*k* = 0.79; 95%CI: 0.48–0.96)^12^.

Attentional problems and emotional reactivity were measured on the respective subscales of the Preschool CBCL^35^; responses were based on caregiver reports. Vision was assessed using the Cardiff Visual Acuity and Contrast Sensitivity tests for binocular vision^15^. These are indicative of the integrity of the visual pathway and central nervous system and, as directly observed neurodevelopmental markers, are unlikely to be affected by cultural influences.

Motor development was assessed against four WHO milestones that are less likely to be affected by recall bias: sitting without support, hands and knees crawling, standing alone and walking alone^17^. Trained staff collected the data on a form with pictures of the relevant child positions and corresponding definitions. Parents were asked to report the age in months and weeks when they first observed or ‘never observed’ the milestones. We assessed the age (in months) at which WHO gross motor milestones were first achieved.

All INTER-NDA assessors were trained centrally according to the study protocol, and, in turn, they trained local assessors who were standardized annually. All assessors were subject to a protocol adherence and reliability assessment after training, and only those with protocol adherence scores in excess of 90% and inter-rater reliability of >0.8 conducted assessments.

The administration of the above tests was supported by a tablet-based data collection and management system, developed for the INTERGROWTH-21st Project^12^. Field staff were unaware of the INTER-NDA domain and total scores for individual children and sites. Data were uploaded onto secure servers as soon as each assessment was completed.

The INTER-NDA was used in Phase I of the INTERGROWTH-21st Project. The results clearly demonstrated that the children of healthy, adequately nourished, well-educated women, recruited from five diverse geographical and cultural study sites, who receive recommended ante-natal care, display consistent similarities at age 2 across a comprehensive set of neurodevelopmental outcomes^16^. International normative values have been established by pooling the data from these samples^8^.

### Data management system

Data were managed with a similar system to that used in Phase I of the INTERGROWTH-21st Project. In brief, demographic, medical history and maternal and newborn clinical data were collected initially on paper forms and then entered locally into an online data management system developed for the INTERGROWTH-21st Project (MedSciNet), which sits on a secure MedSciNet server. Blinded data from the ultrasound machines were transferred directly to the database in Oxford. The anonymized databases are accessible only to designated personnel, including the Bill & Melinda Gates Foundation, as part of a data-sharing agreement. Users from each study site have access, at present, only to their own data; a small number of global administrators have access to all the data on a high-security, encrypted server.

### Statistical analysis

We included all pregnant women (*n* = 3,206) with three or more fetal ultrasound scans between 14 and 37 weeks’ gestation (mean = 4.7 scans) to describe fetal growth patterns (Supplementary Fig. 2). We did not include in the analyses any ultrasound measures taken after 37 weeks’ gestation because of the well-recognized increase in measurement error at term. Sex- and gestational age-specific *z*-scores were estimated for fetal HC and AC using the international INTERGROWTH-21st Fetal Growth Standards^5^. Twenty-three fetal measures (HC = 12 and AC = 11) with *z*-scores >5 or <−5 were excluded from the analysis.

To determine fetal cranial growth trajectories, we constructed models for repeated measurements of HC *z*-scores using finite mixture models^36^—a data-driven approach for linear and non-linear trajectories—to model longitudinal trajectories and to identify members of a specified number of latent classes based on distinct growth patterns. These models capture heterogeneity in growth patterns by minimizing the variability around estimated trajectory means for a specified number of groups. Finite mixture models have been used to identify growth trajectories in children and their relationship to exposures, sleep and health outcomes^37–42^. Group mean growth patterns were modeled using Gaussian distributions by applying a quadratic B-spline with one internal knot placed at the median^43^. A random intercept for each participant was added to highlight growth trajectories relative to the initial ultrasound and prevent grouping participants strictly by size. All mixture modeling was done using the hmle function of the R package lcmm^37,44^.

Posterior group probabilities for models with three to five growth pattern groups were estimated^45^, allowing for a limited number of trajectories that follow clinically recognized patterns of fetal growth. Trajectories were determined using only the fetal measures without considering their associated neurodevelopmental outcomes, to which the statistician responsible (S.A.R.) was blinded at the time of the analysis. The optimal number of groups for each fetal growth measure was selected based on 1) the best model fit using the BIC and 2) the number of participants in the smallest group comprising at least 2.5% of the total sample. Each participant received a posterior probability of being in each group and was then assigned to the definitive group with the highest probability.

Fetal cranial growth trajectories comprised the primary exposures. The trajectory most likely to be adequate (tracking the 50th centile) according to the international INTERGROWTH-21st Fetal Growth Standards^5^ was chosen as the reference group; this trajectory is also the one with the largest number of fetuses.

Outcomes from the neurodevelopmental assessment were based on normative INTER-NDA scores for the six domains^8,12^. Behavioral outcomes were based on the CBCL attentional problems and emotional reactivity scales^35^. Categories of low visual acuity (defined as logMAR >0.4) and high contrast sensitivity (>3%) were based on the Cardiff norms for age 2–3 (ref.^15^).

Height/length, weight, HC and BMI were assessed at age 2; measures were used to estimate age- and sex-standardized *z*-scores based on the WHO Child Growth Standards^7^. Height was considered the primary infant growth outcome. We excluded extreme measurements that were likely to be errors (HC = 1 and height = 7).

As our primary analyses, we used linear regression models to assess the relationships with cognitive, language, fine motor and gross motor domains. The positive and negative affect scores were strongly left- and right-skewed, respectively. The positive affect domain was reverse coded, and both positive and negative scales were modeled as count data using general linear models, using a Poisson distribution and log link function, with a variance correction for over dispersion. Vision outcomes were modeled as binary variables and used Poisson regression as well. The results represent a change in score or RR for each trajectory compared to the reference MGT trajectory. We used linear regression models to assess growth outcomes at age 2 and Cox proportional hazards models to model the age at which gross motor development milestones were first achieved.

We selected covariates suspected a priori to be in the causal pathway, using separate directed acyclic graphs for size and neurodevelopmental outcomes. In all adjusted models, we included the child’s sex and age at developmental assessment, preterm birth, maternal age and education (primary or no schooling, secondary or professional/technical school or any university education) as well as smoking during pregnancy. Age at achieving gross motor development outcome models did not include child age as a covariate.

As secondary sensitivity analyses, we evaluated effect modification by duration of breastfeeding (<7 months and ≥7 months) using stratified models. We constructed models that included both fetal HC and AC growth trajectories to explore whether the observed associations with fetal HC were independent effects or partially due to a relationship with the growth trajectory of the abdominal organs, given the value of measuring fetal AC alone to predict birth weight. In sensitivity analyses, we also ran models without including preterm birth as a covariate and excluded (in separate models) children who had 1) any NICU admission; 2) serious morbidity (cardiovascular, gastrointestinal, hemolytic, serious injury or surgery); 3) at least three infections in a single year requiring antibiotics; and 4) been hospitalized. We also conducted sensitivity analyses 1) including outliers for HC (*n* = 1), height (*n* = 3) and weight (*n* = 1) and 2) excluding children missing one or more questions for fine (*n* = 137) and gross (*n* = 187) motor domains and those missing more than one question for cognitive (*n* = 163) and language (*n* = 147) domains. Finally, we conducted exploratory analyses adjusting by anthropometric measures at birth and age 2.

All analyses were performed using R (v3.6.0, The R Core Team, 2018) and STATA (v15.0, StataCorp, 2017).

## Supplementary Material

The online version contains supplementary material available at https://doi.org/10.1038/s41591-021-01280-2.

Supplementary Information

Supplementary Materials

## Figures and Tables

**Table 1:** Changes in developmental outcomes at 2 years of age associated with fetal cranial growth trajectories relative to MGT trajectory^a^ in the INTERBIO-21st Fetal Study

Outcome	Early faltering (*n* = 257)	*P* value	Late faltering (*n* = 185)	*P* value	Accelerating growth (*n* = 265)	*P* value	Late median growth (*n* = 192)	*P* value
Neurodevelopmental assessment^b^								
Cognitive	−4.12 (−6.45, −1.80)	<0.01	−1.65 (−4.25, 0.95)	0.21	1.53 (−0.72, 3.77)	0.18	1.46 (−1.13, 4.05)	0.27
Language	−7.16 (−10.12, −4.20)	<0.01	−2.22 (−5.53, 1.09)	0.19	3.45 (0.59, 6.32)	0.02	3.48 (0.18, 6.78)	0.04
Fine motor	−3.75 (−5.67, −1.83)	<0.01	−1.45 (−3.60, 0.70)	0.19	0.90 (−0.96, 2.76)	0.34	1.00 (−1.14, 3.15)	0.36
Gross motor	−1.15 (−3.45, 1.16)	0.33	0.93 (−1.64, 3.51)	0.48	0.59 (−1.65, 2.83)	0.60	−1.79 (−4.36, 0.78)	0.17
Positive behavior^c^	0.93 (0.79, 1.08)	0.35	0.89 (0.75, 1.05)	0.17	1.16 (0.98, 1.38)	0.09	1.09 (0.91, 1.31)	0.34
Negative behavior^c^	1.13 (0.99, 1.31)	0.08	1.05 (0.89, 1.23)	0.57	0.84 (0.72, 0.98)	0.03	0.87 (0.72, 1.06)	0.18
Communication and attention subset	1.25 (1.12,1.40)	<0.01	1.09 (0.95, 1.24)	0.21	0.98 (0.88,1.09)	0.68	1.04 (0.92,1.16)	0.55
Child Behavior Questionnaire^b^								
Attentional problems	−0.11 (−0.44, 0.22)	0.51	0.24 (−0.13, 0.61)	0.20	−0.01 (−0.32, 0.31)	0.97	0.19 (−0.17, 0.55)	0.30
Emotional reactivity	0.47 (−0.02, 0.97)	0.06	0.82 (0.27, 1.38)	<0.01	−0.36 (−0.83, 0.12)	0.14	−0.12 (−0.67, 0.43)	0.66
Vision deficits^d^								
Acuity >0.4 logMAR	2.02 (1.61, 2.53)	<0.01	1.62 (1.20, 2.19)	<0.01	0.52 (0.32, 0.85)	0.01	0.57 (0.33, 1.01)	0.05
Contrast sensitivity >33.3%	1.92 (1.44, 2.58)	<0.01	1.49 (1.00, 2.22)	0.05	0.52 (0.29, 0.93)	0.03	0.48 (0.23, 1.02)	0.06
Growth at 2 years of age^b^								
Height *z*-score	−0.48 (−0.64, −0.31)	<0.01	−0.08 (−0.27, 0.11)	0.41	0.27 (0.10, 0.43)	<0.01	0.38 (0.19, 0.56)	<0.01
Weight *z*-score	−0.49 (−0.65, −0.33)	<0.01	0.03 (−0.15, 0.21)	0.73	0.37 (0.22, 0.53)	<0.01	0.27 (0.09, 0.44)	<0.01
HC *z*-score	−0.54 (−0.70, −0.39)	<0.01	0.23 (0.06, 0.41)	0.01	0.64 (0.48, 0.79)	<0.01	0.55 (0.38, 0.72)	<0.01
BMI	−0.39 (−0.63, −0.16)	<0.01	0.14 (−0.13, 0.40)	0.31	0.37 (0.14, 0.60)	<0.01	0.04 (−0.23, 0.30)	0.78
Motor milestones^e^								
Sitting without help	1.11 (0.97, 1.28)	0.12	1.06 (0.91, 1.24)	0.44	0.92 (0.81, 1.06)	0.25	0.94 (0.81, 1.10)	0.46
Crawling	1.24 (1.08, 1.44)	<0.01	0.96 (0.82, 1.12)	0.60	0.89 (0.78, 1.03)	0.12	0.97 (0.83, 1.14)	0.72
Standing with help	1.06 (0.92, 1.21)	0.45	1.03 (0.88, 1.20)	0.75	0.95 (0.83, 1.09)	0.48	1.02 (0.87, 1.19)	0.84
Standing alone	1.12 (0.97, 1.29)	0.11	0.98 (0.84, 1.15)	0.82	0.92 (0.80, 1.05)	0.22	0.92 (0.79, 1.07)	0.29
Walking with help	1.02 (0.88, 1.17)	0.79	0.99 (0.85, 1.16)	0.93	0.95 (0.83, 1.08)	0.44	0.98 (0.84, 1.14)	0.77
Walking alone	1.08 (0.94, 1.24)	0.27	0.95 (0.82, 1.11)	0.55	0.91 (0.79, 1.04)	0.16	0.91 (0.78, 1.06)	0.24

For cognitive, language, motor and positive behavior outcomes, higher scores represent better outcomes. For negative behavior, attentional problems and emotional reactivity, higher scores represent worse outcomes. For visual acuity and contrast sensitivity, higher RRs represent worse visual performance.

a Models include maternal education (3-level) and age at birth, preterm birth and smoking in pregnancy and child sex and age at assessment.

b Adjusted β and 95% CI from multivariable linear regression models.

c Adjusted incidence rate ratio and 95% CI from multivariable Poisson regression models.

d Adjusted RR and 95% CI from multivariable Poisson regression models.

e Adjusted hazard ratio and 95% CI from Cox proportional hazards models. No adjustments to *P* values were made for multiple comparisons.

## Data Availability

Anonymized data will be made available upon reasonable request for academic use and within the limitations of the informed consent. Requests must be made to the corresponding author. Every request will be reviewed by the INTERBIO-21st Consortium Executive Committee. After approval, the researcher will need to sign a data access agreement with the INTERBIO-21st Consortium.
