# Supplementary material for: Fetal cranial growth trajectories are associated with growth and neurodevelopment at 2 years of age: INTERBIO-21st Fetal Study
Source: Nat Med. Author manuscript; Available in PMC 2022 Sep 7. (PMC7613323; doi:10.1038/s41591-021-01280-2)
Supplement: Supplementary Information [file EMS152539-supplement-Supplementary_Information.pdf]

nature portfolio

https://doi.org/10.1038/s41591-021-01280-2

Supplementary information

Fetal cranial growth trajectories are associated with growth and neurodevelopment at 2 years of age: INTERBIO-21st Fetal Study

In the format provided by the authors and unedited

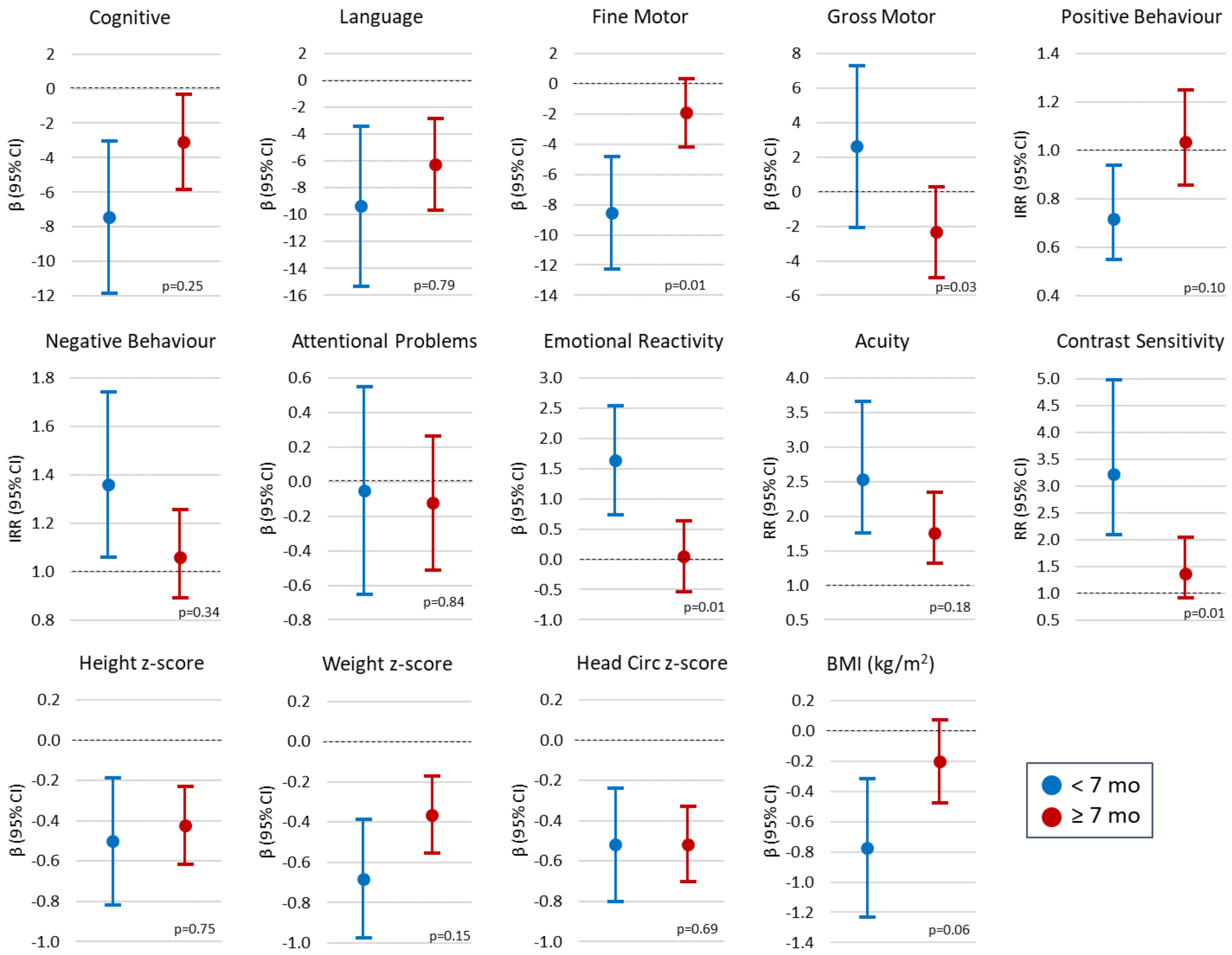

Supplementary Fig.1. Changes in outcomes and 95% confidence intervals at 2 years of age within the Early Faltering growth trajectory, compared with Median growth tracking reference trajectory, stratified by duration of breastfeeding (<7 months, n=71 vs. ≥7

months, n=185) in the INTERBIO-21st Fetal Study. Models include maternal education and age at birth; preterm birth and smoking in pregnancy, and child sex and age at assessment. For cognitive, language, motor, and positive behaviour outcomes, higher scores represent better outcomes. For negative behaviour, attentional problems, and emotional reactivity, higher scores represent worse outcomes. For visual acuity and contrast sensitivity, higher relative risks represent worse visual performance.

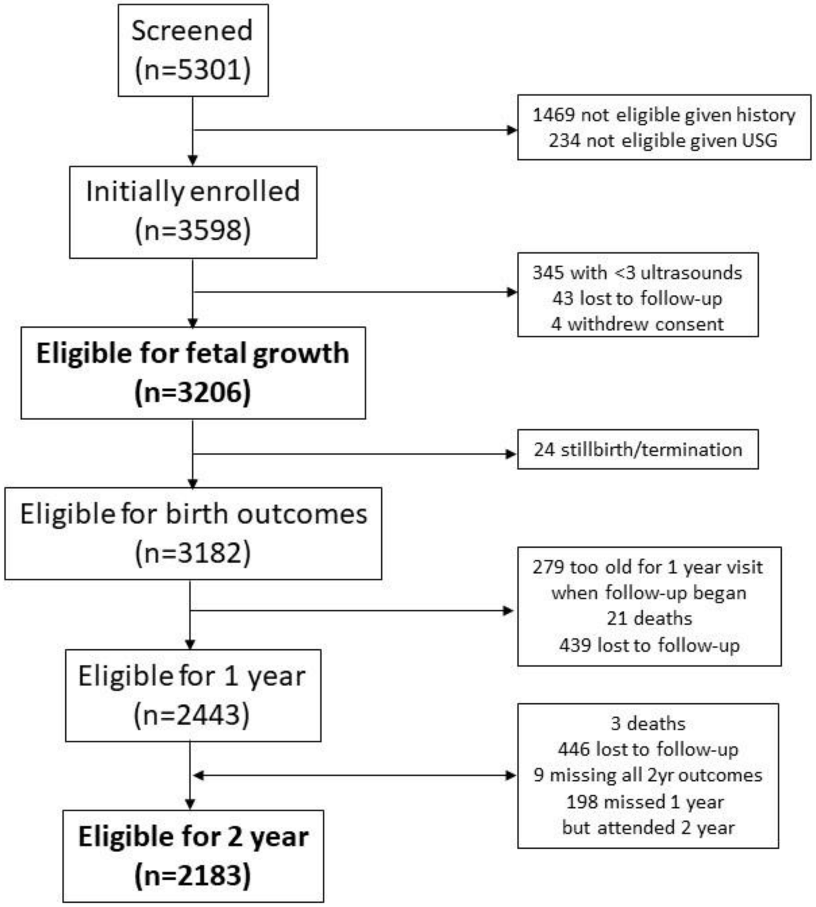

Supplementary Fig.2. INTERBIO-21st Fetal Study participant flow chart and follow-up at 2 years of age.

Supplementary Table 1. Average posterior probabilities for fetal cranial growth trajectory group membership in the INTERBIO-21st Study.

	Median growth	Early faltering growth	Accelerating growth	Late faltering growth	Late median growth
Median growth	0.75	0.06	0.06	0.08	0.06
Early faltering growth	0.14	0.75	0.00	0.11	0.00
Accelerating growth	0.15	0.00	0.75	0.00	0.10
Late faltering growth	0.20	0.14	0.00	0.60	0.06
Late median growth	0.17	0.00	0.11	0.06	0.67
Odds of correct classification	3.8	5.4	6.8	5.5	6.7

Supplementary Table 2. Model fit criteria and minimum number of participants for different numbers of fetal cranial growth trajectory groups in the INTERBIO-21st Study.

Number of groups	AIC	BIC	Min. Participants (%)
4	32901	33053	9.0%
5	32841	33029	8.0%
6	32805	33030	2.5%

AIC = Akaike information criterion; BIC = Bayesian information criterion

Supplementary Table 3. Child medical history at 1 and 2 years in study population followed to 2 years of age (n=2,183).

	1 year, n (%)	2 years, n (%)
Hospitalised at least once	394 (19.8)	249 (11.4)
Total number of days hospitalised[a]	4 (2 – 9)	3 (2 – 7)
Prescription made by health care professional	1780 (81.5)	1992 (91.3)
Antibiotics (≥3 regimens)	375 (18.8)	446 (20.5)
Chromosomal/congenital anomalies	43 (2.2)	35 (1.6)
Otitis media (≥3 episodes) (+ pneumonia/bronchiolitis)	249 (12.5)	295 (13.5)
Parasitosis/diarrhoea/vomiting	148 (7.4)	160 (7.3)
Seizures/cerebral palsy/neurological disorders	13 (0.7)	29 (1.3)
Exanthema/skin disease	485 (24.4)	450 (20.6)
Urinary tract infection/pyelonephritis	9 (0.5)	5 (0.2)
Fever (≥3 episodes of ≥3 days)	210 (10.5)	272 (12.5)
Malaria	3 (0.2)	12 (0.6)
HIV/AIDS	8 (0.4)	4 (0.2)
Meningitis	11 (0.6)	4 (0.2)
Blindness	3 (0.2)	3 (0.1)
Hearing problems	2 (0.1)	4 (0.2)
Asthma	26 (1.3)	47 (2.2)
Cardiovascular problems	10 (0.5)	10 (0.5)
Gastroesophageal reflux	144 (7.2)	33 (1.5)
Injury/trauma	46 (2.3)	96 (4.4)
Any condition requiring surgery	40 (2.0)	48 (2.2%)
Any haemolytic condition	59 (3.0)	48 (2.2)
Any malignancy	3 (0.2)	0 (0.0)
Other infections requiring antibiotics	83 (4.2)	110 (5.1)
Malnutrition/growth problems	53 (2.7)	48 (2.2)
Severe morbidity[b]	281 (12.9)	217 (9.9)

[a] Median (interquartile range)

[b] Severe morbidity includes cardiovascular, gastroesophageal, and haemolytic conditions, as well as injury trauma and any other condition requiring surgery

Supplementary Table 4. Child neurodevelopmental, visual, motor and anthropometric outcomes at 2 years of age, according to fetal cranial growth trajectories in the INTERBIO-21st Fetal Study.

	Early Faltering (n=257) Mean ± SD	Late Faltering (n=185) Mean ± SD	Median Growth Tracking (n=1284) Mean ± SD	Accelerating Growth (n=265) Mean ± SD	Late Median Growth (n=192) Mean ± SD	All participants (n=2183) Mean ± SD
Neurodevelopmental Assessment, scaled score[a]						
Cognitive	65.0 ± 17.2	68.1 ± 18.0	70.6 ± 16.3	72.5 ± 16.8	72.0 ± 16.4	70.1 ± 16.8
Language	51.5 ± 19.9	57.6 ± 21.5	61.3 ± 21.6	65.4 ± 22.2	64.3 ± 22.1	60.6 ± 21.9
Fine motor	84.1 ± 15.1	87.5 ± 15.4	89.5 ± 13.4	90.9 ± 13.0	91.0 ± 13.3	89.0 ± 13.9
Gross motor	79.1 ± 17.5	82.3 ± 14.1	81.3 ± 16.2	82.3 ± 15.9	80.3 ± 17.1	81.2 ± 16.2
Positive behaviour	76.5 ± 25.4	75.3 ± 25.8	78.6 ± 24.3	81.5 ± 23.5	80.1 ± 22.6	78.5 ± 24.3
Negative behaviour	29.7 ± 28.7	28.1 ± 28.6	25.8 ± 27.7	21.5 ± 25.1	23.4 ± 29.5	25.7 ± 27.8
Communication and Attention subset[b]	2.9 ± 2.3	2.5 ± 2.1	2.2 ± 1.7	2.2 ± 1.7	2.3 ± 1.7	2.3 ± 1.8
Behavioural Assessment, raw score[c]						
Attentional problems	3.7 ± 2.2	4.2 ± 2.3	3.9 ± 2.3	3.9 ± 2.3	4.3 ± 2.4	3.9 ± 2.3
Emotional reactivity	5.9 ± 3.6	6.4 ± 3.6	5.7 ± 3.5	5.4 ± 3.7	5.7 ± 3.5	5.7 ± 3.6
Measurements at 2 years of age						
Height (z-score)	-1.00 ± 1.19	-0.44 ± 1.18	-0.33 ± 1.26	0.01 ± 1.23	0.12 ± 1.10	-0.34 ± 1.26
Weight (z-score)	-0.63 ± 1.20	0.05 ± 1.27	0.04 ± 1.21	0.48 ± 1.16	0.36 ± 1.10	0.04 ± 1.23
Head circumference (z-score)	-0.82 ± 1.15	0.09 ± 1.26	-0.11 ± 1.18	0.59 ± 1.05	0.47 ± 1.04	-0.04 ± 1.22
BMI (kg/m^2)	15.7 ± 1.6	16.4 ± 1.7	16.2 ± 1.8	16.7 ± 1.6	16.4 ± 1.6	16.2 ± 1.7
Motor milestones, months						
Sitting up without help	5.9 ± 1.5	6.0 ± 1.4	6.0 ± 1.3	6.1 ± 1.4	6.1 ± 1.3	6.0 ± 1.3
Crawling	7.7 ± 1.7	8.3 ± 2.3	8.2 ± 1.9	8.5 ± 1.9	8.4 ± 1.5	8.2 ± 1.9
Standing with help	9.2 ± 1.6	9.3 ± 2.0	9.2 ± 1.7	9.3 ± 1.9	9.2 ± 1.5	9.2 ± 1.7
Standing alone	11.2 ± 1.6	11.5 ± 1.9	11.4 ± 1.8	11.6 ± 1.7	11.6 ± 1.7	11.4 ± 1.8
Walking with help	10.2 ± 1.7	10.3 ± 2.2	10.2 ± 1.6	10.3 ± 1.9	10.3 ± 1.5	10.2 ± 1.7
Walking alone	12.7 ± 2.0	12.9 ± 2.2	12.8 ± 2.0	12.9 ± 2.2	13.0 ± 2.0	12.8 ± 2.1

	n (%)	n (%)	n (%)	n (%)	n (%)	n (%)
Vision Deficits						
Acuity >0.4 LogMAR	78 (34.1)	38 (23.6)	161 (13.9)	16 (6.7)	12 (6.8)	305 (15.5)
Contrast Sensitivity >33.3%	52 (23.2)	24 (15.4)	118 (10.5)	12 (5.2)	7 (4.2)	213 (11.2)

[a]Scaled scores for cognitive, language, motor, and positive and negative behaviour domains range from 0-100.

[b]Communication and attention subset score represents a sum of 18 items on the neurodevelopmental assessment.

[c]Raw scores for attentional problems and emotional reactivity represent the sum of 5 and 9 questions, respectively.

Supplementary Table 5. Adjusted outcomes[a] at 2 years of age stratified by duration of breastfeeding (less than 7 months vs. 7 months or longer) in the INTERBIO-21st Study.

Outcome	Breastfeeding	Early Faltering	Late Faltering	Accelerating Growth	Late Median Growth Tracking
Neurodevelopmental Assessment[b]					
Cognitive	<7 mo	-7.44 (-11.85, -3.04)*	2.67 (-2.58, 7.92)	-0.50 (-4.42, 3.43)	1.61 (-2.91, 6.12)
	≥7 mo	-3.06 (-5.81, -0.30)*	-2.74 (-5.75, 0.26)#	2.42 (-0.33, 5.17)#	1.28 (-1.89, 4.46)
	p_{int}	0.25	0.12	0.19	0.91
Language	<7 mo	-9.35 (-15.31, -3.39)*	2.98 (-4.13, 10.09)	4.25 (-1.06, 9.57)	4.28 (-1.82, 10.39)
	≥7 mo	-6.27 (-9.69, -2.85)*	-3.58 (-7.31, 0.15)#	2.94 (-0.47, 6.35)#	2.89 (-1.05, 6.82)
	p_{int}	0.79	0.16	0.90	0.85
Fine motor	<7 mo	-8.54 (-12.25, -4.83)*	1.72 (-2.70, 6.15)	-1.33 (-4.66, 2.00)	1.69 (-2.11, 5.49)
	≥7 mo	-1.90 (-4.16, 0.36)#	-2.35 (-4.82, 0.11)#	1.94 (-0.31, 4.20)#	0.64 (-1.96, 3.24)
	p_{int}	0.01	0.16	0.09	0.89
Gross Motor	<7 mo	2.63 (-2.05, 7.30)	5.61 (0.03, 11.19)*	-0.81 (-5.00, 3.38)	-1.30 (-6.09, 3.49)
	≥7 mo	-2.34 (-4.97, 0.29)#	-0.54 (-3.41, 2.33)	1.61 (-1.02, 4.24)	-1.82 (-4.85, 1.21)
	p_{int}	0.03	0.09	0.21	0.93
Positive Behaviour[c]	<7 mo	0.72 (0.55, 0.94)*	0.90 (0.64, 1.26)	0.87 (0.66, 1.16)	0.95 (0.68, 1.32)
	≥7 mo	1.03 (0.86, 1.25)	0.91 (0.75, 1.10)	1.34 (1.07, 1.67)*	1.15 (0.93, 1.42)
	p_{int}	0.10	0.69	0.01	0.19
Negative Behaviour[c]	<7 mo	1.36 (1.06, 1.74)*	1.00 (0.74, 1.34)	1.01 (0.77, 1.32)	1.00 (0.72, 1.39)
	≥7 mo	1.06 (0.89, 1.26)	1.05 (0.87, 1.26)	0.77 (0.63, 0.93)*	0.83 (0.65, 1.05)
	p_{int}	0.34	0.88	0.06	0.22
Communication and Attention subset[c]	<7 mo	1.42 (1.17, 1.73)*	0.90 (0.69, 1.18)	1.05 (0.87, 1.28)	1.10 (0.90, 1.35)
	≥7 mo	1.20 (1.05, 1.38)*	1.15 (0.99, 1.34)#	0.94 (0.83, 1.06)	1.00 (0.87, 1.15)
	p_{int}	0.34	0.13	0.27	0.34
Attention Problems	<7 mo	-0.05 (-0.65, 0.55)	0.44 (-0.29, 1.17)	-0.53 (-1.07, 0.00)#	-0.18 (-0.80, 0.44)
	≥7 mo	-0.12 (-0.51, 0.27)	0.22 (-0.21, 0.64)	0.23 (-0.16, 0.62)	0.34 (-0.11, 0.78)
	p_{int}	0.84	0.63	0.02	0.20

Emotional Reactivity	<7 mo	1.64 (0.73, 2.54)*	0.79 (-0.31, 1.89)	-0.79 (-1.59, 0.02)#	-1.01 (-1.94, -0.07)*
	≥7 mo	0.05 (-0.54, 0.63)	0.78 (0.14, 1.42)*	-0.10 (-0.69, 0.48)	0.33 (-0.34, 1.00)
	p_{int}	0.01	0.95	0.17	0.02
Vision Deficits[d]					
Acuity >0.4 LogMAR	<7 mo	2.53 (1.76, 3.66)*	1.06 (0.53, 2.12)	0.35 (0.13, 0.93)*	0.75 (0.34, 1.64)
	≥7 mo	1.76 (1.32, 2.35)*	1.76 (1.26, 2.46)*	0.62 (0.36, 1.09)#	0.48 (0.21, 1.06)#
	p_{int}	0.18	0.21	0.37	0.38
Contrast sensitivity >33.3%	<7 mo	3.22 (2.09, 4.97)*	0.85 (0.34, 2.09)	0.26 (0.06, 1.06)#	0.56 (0.19, 1.70)
	≥7 mo	1.36 (0.91, 2.04)	1.65 (1.07, 2.55)*	0.69 (0.37, 1.29)	0.46 (0.17, 1.24)
	p_{int}	0.01	0.35	0.23	0.64
Growth at 2 years of age[b]					
Height z-score	<7 mo	-0.50 (-0.82, -0.19)*	-0.05 (-0.44, 0.33)	0.29 (0.00, 0.58)*	0.37 (0.04, 0.70)*
	≥7 mo	-0.42 (-0.62, -0.23)*	-0.08 (-0.29, 0.13)	0.24 (0.05, 0.44)*	0.35 (0.13, 0.57)*
	p_{int}	0.75	0.64	0.95	1.00
Weight z-score	<7 mo	-0.68 (-0.97, -0.39)*	-0.06 (-0.42, 0.30)	0.35 (0.09, 0.62)*	0.23 (-0.08, 0.53)
	≥7 mo	-0.36 (-0.55, -0.17)*	0.08 (-0.13, 0.29)	0.35 (0.16, 0.54)*	0.24 (0.02, 0.45)*
	p_{int}	0.15	0.70	0.70	0.73
Head circumference z-score	<7 mo	-0.52 (-0.80, -0.24)*	0.09 (-0.25, 0.43)	0.68 (0.43, 0.94)*	0.51 (0.23, 0.80)*
	≥7 mo	-0.52 (-0.70, -0.33)*	0.28 (0.07, 0.48)*	0.60 (0.41, 0.79)*	0.55 (0.33, 0.76)*
	p_{int}	0.69	0.50	0.95	0.56
BMI	<7 mo	-0.77 (-1.23, -0.32)*	-0.13 (-0.69, 0.43)	0.29 (-0.13, 0.71)	-0.11 (-0.58, 0.37)
	≥7 mo	-0.20 (-0.48, 0.07)	0.25 (-0.05, 0.55)#	0.36 (0.08, 0.63)*	0.04 (-0.27, 0.36)
	p_{int}	0.06	0.25	0.61	0.44
Motor Milestones[e]					
Sitting Alone	<7 mo	1.17 (0.90, 1.52)	1.15 (0.84, 1.57)	1.04 (0.82, 1.31)	1.04 (0.79, 1.37)
	≥7 mo	1.07 (0.91, 1.27)	1.03 (0.86, 1.24)	0.86 (0.73, 1.02)#	0.89 (0.74, 1.08)
	p_{int}	0.36	0.53	0.19	0.40

Crawling	<7 mo	1.42 (1.09, 1.86)*	1.05 (0.76, 1.45)	0.88 (0.69, 1.13)	0.99 (0.75, 1.32)
	≥7 mo	1.14 (0.96, 1.35)	0.94 (0.78, 1.13)	0.91 (0.76, 1.08)	0.98 (0.81, 1.19)
	p_{int}	0.17	0.77	1.00	0.93
Standing with help	<7 mo	1.10 (0.85, 1.43)	1.08 (0.79, 1.49)	0.94 (0.74, 1.19)	0.96 (0.73, 1.26)
	≥7 mo	1.03 (0.87, 1.22)	1.01 (0.84, 1.20)	0.95 (0.80, 1.12)	1.04 (0.86, 1.26)
	p_{int}	0.67	0.61	0.85	0.82
Standing alone	<7 mo	1.20 (0.93, 1.56)	1.05 (0.77, 1.44)	0.80 (0.63, 1.02)#	0.79 (0.60, 1.03)#
	≥7 mo	1.08 (0.91, 1.27)	0.97 (0.81, 1.16)	0.97 (0.83, 1.15)	0.99 (0.82, 1.20)
	p_{int}	0.47	0.57	0.28	0.33
Walking with help	<7 mo	1.16 (0.89, 1.51)	0.96 (0.69, 1.32)	0.97 (0.76, 1.23)	0.92 (0.70, 1.21)
	≥7 mo	0.97 (0.81, 1.14)	1.01 (0.84, 1.21)	0.93 (0.79, 1.10)	1.02 (0.84, 1.23)
	p_{int}	0.17	0.91	0.78	0.54
Walking alone	<7 mo	1.22 (0.94, 1.58)	1.12 (0.81, 1.53)	0.86 (0.68, 1.09)	0.88 (0.67, 1.15)
	≥7 mo	1.03 (0.87, 1.21)	0.92 (0.77, 1.11)	0.92 (0.78, 1.08)	0.92 (0.76, 1.11)
	p_{int}	0.22	0.24	0.68	0.85

For cognitive, language, motor and positive behaviour outcomes, higher scores represent better outcomes. For negative behaviour. Attentional problems and emotional reactivity, higher scores represent worse outcomes. For visual acuity and contrast sensitivity, higher relative risks represent worse visual performance.

[a] Median Growth Tracking trajectory is the reference group. Models include maternal education and age at birth; preterm birth and smoking in pregnancy, and child sex and age at assessment.

[b] Adjusted β and 95% CI from multivariable linear regression models

[c] Adjusted Incidence Rate Ratio and 95% CI from multivariable Poisson regression models

[d] Adjusted Relative Risk and 95% CI from multivariable Poisson regression models

[e] Adjusted Hazard Ratio and 95% CI from Cox proportional hazards models

[#]p<0.1; [*]p<0.05, no adjustments for multiple comparisons were made

Supplementary Table 6. Description of the maternal study cohort and comparison between those followed at 2 years of age (n=2,183), those lost to follow-up[a] (n=1,023) and all participants (n=3,206) in the INTERBIO-21st Fetal Study.

	Completed age 2 visit (n=2183)	Lost to follow-up[a] (n=1023)	All participants (n=3206)
Maternal age at delivery[b]	29.4 ± 5.2	28.9 ± 5.7	29.2 ± 5.4
Height (cm)[b]	160.3 ± 7.5	159.0 ± 7.5	159.9 ± 7.5
Married/living as married, n (%)	1927 (88.3)	817 (79.9)	2744 (85.6)
University education, n (%)	929 (42.6)	314 (30.7)	1243 (38.8)
Work outside the home, n (%)	1553 (71.2)	707 (69.1)	2260 (70.5)
Early pregnancy weight (kg)[b]	64.0 ± 12.8	62.1 ± 13.5	63.4 ± 13.1
Early pregnancy BMI (kg/m^2)[b]	24.8 ± 4.3	24.4 ± 4.5	24.7 ± 4.3
Smoking during pregnancy, n (%)	89 (4.1)	73 (7.1)	162 (5.1)
Alcohol during pregnancy, n (%)	49 (2.2)	27 (2.6)	76 (2.4)
Multiparous, n (%)	1269 (58.2)	636 (62.2)	1905 (59.4)
Caesarean section, n (%)	911 (41.7)	373 (36.5)	1284 (40.1)
Gestational diabetes, n (%)	164 (7.5)	33 (3.2)	197 (6.1)

[a] Children lost to follow-up before evaluation at 2 years of age.
[b] Data are means ± standard deviation, BMI=body mass index.

Supplementary Table 7. Child measurements and characteristics, and comparison between study population (n=2,183), those lost to follow-up[a] (n=1,023) and all participants (n=3,206) in the INTERBIO-21st Fetal Study.

	Completed all visits (n=2183)	***Lost to follow-up[a] (n=1023)***	***All participants*** (n=3206)
Birthweight, g[b]	3129 ± 511	3023 ± 573	3095 ± 533
Birth length, cm[b]	48.7 ± 2.3	48.4 ± 2.8	48.6 ± 2.5
Birth head circumference, cm[b]	33.9 ± 1.5	33.5 ± 1.8	33.8 ± 1.6
Gestational age at delivery, weeks[b]	39.1 ± 1.8	38.8 ± 2.3	39.0 ± 2.0
Preterm, <37 weeks' gestation, n (%)	204 (9.3)	142 (13.9)	346 (10.8)
Very preterm, <34 weeks' gestation, n (%)	36 (1.7)	40 (3.9)	76 (2.4)
Boys, n (%)	1139 (52.2)	520 (50.8)	1659 (51.8)
Congenital anomaly	37 (1.7)	15 (1.5)	52 (1.6)
Neonatal Intensive Care Unit stay, n (%)	205 (9.4)	165 (16.1)	370 (11.5)
Breastfed for ≥7 months, n (%)	1548 (71.0)	MD[d]	1775 (70.8)
Age at weaning (months)[c]	6 (5 - 6)	6 (5 - 6)	6 (5 - 6)

[a] Children lost to follow-up before evaluation at 2 years of age.
[b] Means ± standard deviation.
[c] Median (interquartile range).
[d] MD = missing data for many infants lost to follow-up.
