# Supplementary material for: Fetal cranial growth trajectories are associated with growth and neurodevelopment at 2 years of age: INTERBIO-21st Fetal Study
Source: Nat Med. Author manuscript; Available in PMC 2022 Sep 7. (PMC7613323; doi:10.1038/s41591-021-01280-2)
Supplement: Supplementary Materials [file EMS152539-supplement-Supplementary_Materials.pdf]

nature research

Corresponding author(s): Jose Villar

Last updated by author(s): 2021.01.05

Reporting Summary

Nature Research wishes to improve the reproducibility of the work that we publish. This form provides structure for consistency and transparency in reporting. For further information on Nature Research policies, see our Editorial Policies and the Editorial Policy Checklist.

Statistics

For all statistical analyses, confirm that the following items are present in the figure legend, table legend, main text, or Methods section.

n/a	Confirmed	
☐	☒	The exact sample size (*n*) for each experimental group/condition, given as a discrete number and unit of measurement
☐	☒	A statement on whether measurements were taken from distinct samples or whether the same sample was measured repeatedly
☐	☒	The statistical test(s) used AND whether they are one- or two-sided<br>*Only common tests should be described solely by name; describe more complex techniques in the Methods section.*
☐	☒	A description of all covariates tested
☐	☒	A description of any assumptions or corrections, such as tests of normality and adjustment for multiple comparisons
☐	☒	A full description of the statistical parameters including central tendency (e.g. means) or other basic estimates (e.g. regression coefficient) AND variation (e.g. standard deviation) or associated estimates of uncertainty (e.g. confidence intervals)
☐	☒	For null hypothesis testing, the test statistic (e.g. *F*, *t*, *r*) with confidence intervals, effect sizes, degrees of freedom and *P* value noted<br>*Give P values as exact values whenever suitable.*
☒	☐	For Bayesian analysis, information on the choice of priors and Markov chain Monte Carlo settings
☒	☐	For hierarchical and complex designs, identification of the appropriate level for tests and full reporting of outcomes
☒	☐	Estimates of effect sizes (e.g. Cohen's *d*, Pearson's *r*), indicating how they were calculated

Our web collection on statistics for biologists contains articles on many of the points above.

Software and code

Policy information about availability of computer code

Data collection	Data collection forms and an electronic data entry system specifically developed for the study (www.interbio21.org.uk) and were collected initially on paper forms and then entered locally into an on-line data management system, developed for the INTERGROWTH-21st Project (MedSciNet, London, UK), which sits on a secure MedSciNet server.
Data analysis	All analyses were performed using R (version 3.6.0, The R Core Team 2018. Vienna, Austria) and STATA (version 15.0, StataCorp. 2017. College Station, Texas).

For manuscripts utilizing custom algorithms or software that are central to the research but not yet described in published literature, software must be made available to editors and reviewers. We strongly encourage code deposition in a community repository (e.g. GitHub). See the Nature Research guidelines for submitting code & software for further information.

Data

Policy information about availability of data

All manuscripts must include a data availability statement. This statement should provide the following information, where applicable:

- Accession codes, unique identifiers, or web links for publicly available datasets
- A list of figures that have associated raw data
- A description of any restrictions on data availability

The anonymised databases are only accessible to designated personnel, including the Bill & Melinda Gates Foundation as part of a data sharing agreement.

Field-specific reporting

Please select the one below that is the best fit for your research. If you are not sure, read the appropriate sections before making your selection.

☐ Life sciences ☒ Behavioural & social sciences ☐ Ecological, evolutionary & environmental sciences

For a reference copy of the document with all sections, see nature.com/documents/nr-reporting-summary-flat.pdf

Behavioural & social sciences study design

All studies must disclose on these points even when the disclosure is negative.

Study description	This study is a prospective cohort of pregnant mothers and their children followed until two years of age. Quantitative assessments of fetal growth and neurodevelopment and growth were analyzed.
Research sample	The research sample is derived from a multi-national pregnancy cohort (n=3,598), conducted, between 2012 and 2019, at six sites: Pelotas (Brazil), Nairobi (Kenya), Karachi (Pakistan), Soweto (South Africa), Mae Sot (Thailand) and Oxford (UK). The sample is representative of singleton births in these countries. Participants were 29.4 (+/- 5.2) years of age, 88% were married and 43% had a university education.
Sampling strategy	In the INTERBIO-21st Fetal Study, we enrolled women who initiated antenatal care before 14 weeks' gestation, irrespective of their risk profile for adverse pregnancy/neonatal outcomes, and monitored their pregnancies to delivery. Thereafter, the health, growth and development of their children were monitored up until age 2.
Data collection	Researchers were blind to fetal cranial growth phenotypes used in our analyses. Ultrasound data were collected using standardized methods and identical ultrasound equipment at all sites. Growth measurements were made at birth, at age 1 and 2, the infants' weight, length, head circumference, mid-upper arm circumference, triceps skinfold thickness and subscapular skinfold thickness were measured following WHO protocols, and their age- and sex-specific z-scores and centiles were compared to the WHO Child Growth Standards. We assessed neurodevelopment at age 2 using the INTERGROWTH-21st Neurodevelopmental Assessment (INTER-NDA), a comprehensive, objective, standardised tool, targeted at children aged 22-30 months, which measures multiple dimensions of early development using a combination of directly administered, concurrently observed and caregiver reported items. Attentional problems and emotional reactivity were measured on the respective subscales of the Preschool Child Behavior Checklist. Responses were based on caregiver reports. Vision was assessed using the Cardiff Visual Acuity and Contrast Sensitivity tests for binocular vision.
Timing	Data were collected between 2012 and 2019 from pregnancy through age two of the child.
Data exclusions	Exclusion criteria were pre-established. he only inclusion criteria at study entry were: maternal age ≥ 18 years, body mass index (BMI) <35 kgm2 (to facilitate ultrasound scanning of the fetus), natural conception, and singleton pregnancy. All other women were eligible to participate after giving written informed consent.
Non-participation	There were 43 participants lost to follow up and 4 that withdrew consent during pregnancy. An additional 439 participants were lost to follow up prior to their one year visit, and 446 participants were lost to follow up prior to their two year visit.
Randomization	Participants were allocated to groups based on the trajectories of fetal head circumference growth throughout pregnancy using finite mixture models, a data-driven approach for linear and non-linear trajectories, to model longitudinal trajectories and to identify members of a specified number of latent classes based on distinct growth patterns.

Reporting for specific materials, systems and methods

We require information from authors about some types of materials, experimental systems and methods used in many studies. Here, indicate whether each material, system or method listed is relevant to your study. If you are not sure if a list item applies to your research, read the appropriate section before selecting a response.

Materials & experimental systems

n/a	Involved in the study	
☒	☐	Antibodies
☒	☐	Eukaryotic cell lines
☒	☐	Palaeontology and archaeology
☒	☐	Animals and other organisms
☐	☒	Human research participants
☒	☐	Clinical data
☒	☐	Dual use research of concern

Methods

n/a	Involved in the study	
☒	☐	ChIP-seq
☒	☐	Flow cytometry
☒	☐	MRI-based neuroimaging

Human research participants

Policy information about studies involving human research participants

Population characteristics	See above
Recruitment	Pregnant women were recruited from hospitals at six sites: Pelotas (Brazil), Nairobi (Kenya), Karachi (Pakistan), Soweto (South Africa), Mae Sot (Thailand) and Oxford (UK).
Ethics oversight	The INTERBIO-21st Study and its ancillary studies were approved by the Oxfordshire Research Ethics Committee "C" (reference: 08/H0606/139), the research ethics committees of the individual participating institutions, as well as the corresponding regional health authorities where the project was implemented.

Note that full information on the approval of the study protocol must also be provided in the manuscript.
